# Glycoprotein Nonmetastatic Melanoma B (Gpnmb)-Positive Macrophages Contribute to the Balance between Fibrosis and Fibrolysis during the Repair of Acute Liver Injury in Mice

**DOI:** 10.1371/journal.pone.0143413

**Published:** 2015-11-23

**Authors:** Kotaro Kumagai, Kazuaki Tabu, Fumisato Sasaki, Yoichiro Takami, Yuko Morinaga, Seiichi Mawatari, Shinichi Hashimoto, Shiroh Tanoue, Shuji Kanmura, Tsutomu Tamai, Akihiro Moriuchi, Hirofumi Uto, Hirohito Tsubouchi, Akio Ido

**Affiliations:** 1 Digestive and Lifestyle Diseases, Department of Human and Environmental Sciences, Kagoshima University Graduate School of Medical and Dental Sciences, Kagoshima, Japan; 2 Pharmaceutical Care and Health Sciences, School of Pharmacy, Shujitsu University, Okayama, Japan; 3 Center for Digestive and Liver diseases, Miyazaki Medical Center Hospital, Miyazaki, Japan; 4 Kagoshima City Hospital, Kagoshima, Japan; RWTH Aachen, GERMANY

## Abstract

**Background and aims:**

Glycoprotein nonmetastatic melanoma B (Gpnmb), a transmembrane glycoprotein that is expressed in macrophages, negatively regulates inflammation. We have reported that Gpnmb is strongly expressed in the livers of rats fed a choline-deficient, L-amino acid-defined (CDAA) diet. However, the role of macrophage-expressed Gpnmb in liver injury is still unknown. This study aimed to clarify the characteristics of infiltrating macrophages that express Gpnmb, and the involvement of Gpnmb in the repair process in response to liver injury.

**Methods:**

C57BL/6J, DBA/2J [DBA] and DBA/2J-Gpnmb^+^ [DBA-g+] mice were treated with a single intraperitoneal injection of carbon tetrachloride (CCl_4_) at a dose of 1.0 mL/kg body weight. Mice were sacrificed at predetermined time points, followed by measurement of serum alanine aminotransferase (ALT) levels and histological examination. Expression of Gpnmb, pro-/anti-inflammatory cytokines, and profibrotic/antifibrotic factors were examined by quantitative RT-PCR and/or Western blotting. Immunohistochemistry, fluorescent immunostaining and flow cytometry were used to determine the expression of Gpnmb, CD68, CD11b and α-SMA, phagocytic activity, and the presence of apoptotic bodies. We used quantitative RT-PCR and ELISA to examine TGF-β and MMP-13 expression and the concentrations and supernatants of isolated infiltrating hepatic macrophages transfected with siGpnmb.

**Results:**

In C57BL/6J mice, serum ALT levels increased at two days after CCl_4_ injection and decreased at four days. Gpnmb expression in the liver was stimulated four days after CCl_4_ injection. Histological examination and flow cytometry showed that Gpnmb-positive cells were almost positive for CD68-positive macrophages, contained engulfed apoptotic bodies and exhibited enhanced phagocytic activity. Isolated infiltrating hepatic macrophages transfected with siGpnmb showed high MMP-13 secretion. There was no significant difference in the magnitude of CCl_4_-induced liver injury between DBA-g+ and DBA mice. However, hepatic MMP-13 expression, as well as α-SMA expression and collagen production, increased significantly in DBA-g+ compared with DBA mice.

**Conclusions:**

Gpnmb-positive macrophages infiltrate the liver during the recovery phase of CCl_4_–induced acute liver injury and contribute to the balance between fibrosis and fibrolysis in the repair process following acute liver injury.

## Introduction

Acute liver failure, which is associated with high mortality, is caused by massive short-term cell death. Since liver regeneration following massive liver necrosis is also impaired, most investigations have focused on the proliferation of hepatocytes. Several investigators have recently reported that non-parenchymal cells, including hepatic stellate cells, sinusoidal endothelial cells and hepatic macrophages, play an important role in the repair of injured livers [1–6]. Hepatic macrophages are involved in the pathogenesis of liver injury as well as in hepatic homeostasis; both resident and infiltrating hepatic macrophages play a vital role in initiating inflammation in response to hepatic injury and in inducing fibrogenesis, and in resolving inflammation and fibrosis [7]. Macrophages exhibit diverse phenotypes by the time of the hepatic repair process. They can be broadly classified as M1 (classical) or M2 (alternative), with the M2 phenotype particularly associated with wound healing [8–9]. However, wound macrophages, recruited to wounds and other sites of tissue injury, exhibit both phenotypes, which change with time and which partially reflect the phenotypes of their monocyte precursors [10]. In an animal model of hepatic fibrosis, hepatic macrophages were implicated in promoting liver fibrosis, and also played a pivotal role in fibrosis regression [11]. Thus, the diversity of macrophage phenotypes and roles make it difficult to clarify the involvement of hepatic macrophages in the repair process in injured livers.

Originally described in melanoma cells, glycoprotein nonmetastatic melanoma B (Gpnmb), also known as osteoactivin and dendritic cell-associated heparin sulfate proteoglycan-dependent integrin ligand, is a heavily N-glycosylated type I transmembrane domain protein with a short cytoplasmic domain containing an endosomal sorting motif [12–14]. Gpnmb is expressed in numerous cell types, including dendritic cells, macrophages, retinal pigment epithelial cells, osteoblasts and osteoclasts [13–19]. Recent studies have demonstrated that Gpnmb expressed in macrophages functions as a feedback regulator of proinflammatory responses [20], and that binding of Gpnmb on antigen-presenting cells to syndecan-4 on activated T cells inhibits T-cell activation [21–22]. Thus, Gpnmb expressed on antigen-presenting cells may negatively regulate inflammation. Additionally, Gpnmb is involved in osteoblast maturation, and is also associated with motor neuron survival [13, 23]. We previously isolated the gene encoding Gpnmb and demonstrated that it was differentially expressed in the livers of rats fed a choline-deficient, L-amino acid-defined diet [24]. We also found that transgenic Gpnmb expression attenuates the development of hepatic fibrosis [25]. Furthermore, a recent report has demonstrated that Gpnmb is expressed in macrophages infiltrating into injured liver tissues [18]. These findings suggest a pathophysiologic role for Gpnmb in various disorders, including liver injury. However, the specific nature of Gpnmb involvement in liver injury remains unknown. In this study, we focused on the role of infiltrating hepatic macrophages, especially those expressing Gpnmb, during the repair process in injured livers in a mouse model of acute liver injury.

## Materials and methods

### Mice

Specific pathogen-free, C57BL/6J mice were sourced from Kyudo (Kumamoto, Japan). DBA Gpnmb mutant mice (DBA/2J: DBA) and DBA Gpnmb wild-type mice (DBA/2J-Gpnmb^*+*^: DBA-g+) were obtained from Jackson Laboratory (Bar Harbor, ME, USA). All animal experimental procedures were approved by the institutional animal care and use committees of Kagoshima University (Permit Numbers: MD08103, MD09031), and were performed in accordance with these committees’ guidelines for animal experiments. We monitored the health of mice daily and euthanized them by cervical dislocation under anesthesia when they demonstrated distress during experiments. All efforts were made to minimize animal suffering.

### A Model of Acute Liver injury

In our acute liver injury model, mice were treated with a single intraperitoneal injection of carbon tetrachloride (CCl_4_) at a dose of 1.0 mL/kg body weight. We sacrificed C57BL/6J mice at 0, 2, and 4 days after CCl_4_ injection, and DBA and DBA-g+ mice at 0, 2, 4, 6, and 8 days after CCl_4_ injection under sodium pentobarbital anesthesia.

### A model of macrophage depletion during the recovery phase after acute liver injury

On the 2nd day after CCl_4_ injection, macrophage depletion was achieved using 200 μl of a clodronate (dichloromethylene disphosphonate)–containing liposome suspension in PBS via intraperitoneal (IP) injection (clodronate was a gift of Roche diagnostics, GmbH, Mannheim, Germany, and was encapsulated in liposomes as described) [26]. Mice were sacrificed at 0, 2, 4, and 6 days after CCl_4_ injection under sodium pentobarbital anesthesia.

### Histological analysis and Alanine Aminotransferase

Hematoxylin-eosin and Sirius red staining were performed according to standard protocols. For immunohistochemical analysis, liver specimens were fixed in 10% buffered formalin or 4% paraformaldehyde and incubated with rat anti-mouse F4/80 (Clone: A3-1, AbD Serotec, Raleigh, NC, USA), rat anti-mouse CD68 (Clone: FA-11, AbD Serotec), goat anti-mouse Gpnmb (Clone: #297310, R&D Systems, Minneapolis, MN, USA), and monoclonal antibody against α-SMA (Clone: E184, Merck Millipore, Billerica, MA, USA). For immunofluorescent staining, paraffin sections were incubated with rat anti-mouse CD68 (Clone: FA-11, AbD Serotec) and goat anti-mouse Gpnmb (Clone: #297310, R&D Systems). These samples then imaged with fluorescent microscopy. The terminal deoxynucleotidyl transferase–mediated deoxyuridine triphosphate nick-end labeling assay (Promega, Madison, WI, USA) was performed on paraffin liver sections according to the manufacturer’s instructions. All samples undergoing immunofluorescent staining were labeled with ProLongR Gold Antifade Mountant with DAPI (Thermo Fisher Scientific Inc., Grand Island, NY, USA). Serum levels of alanine aminotransferase (ALT) were measured with a commercial kit (SRL, Tokyo, Japan).

### Assessment of collagen deposition and activation of stellate cells

Liver sections at 6 and 8 days after CCl_4_ injection in DBA and DBA-g+ mice were analyzed using Sirius red and α-SMA staining to evaluate collagen deposition and activation of stellate cells, respectively. Five fields were randomly selected from each left lobe per sample, and samples from 5 mice in each group were examined. Thus, a total of 25 fields were analyzed for each group. The ratios of the Sirius red- and α-SMA -stained areas to the total area were quantified by image analysis. A BZ-9000 microscope (KEYENCE, Osaka, Japan) was used to capture and analyze the fields at x200 magnification. Image analysis was performed using Quick Grain Standard ver. 5.0.3. software (Inotech, Hiroshima, Japan).

### RNA isolation and real-time PCR

Total RNA was extracted from frozen liver tissues or cells using the Trizol reagent (Invitrogen, Carlsbad, CA). RNA purity was checked using a spectrophotometer. A260/A280 ratios were 1.9–2.1. First-strand cDNA was synthesized from 100 or 500 ng of total RNA using a PrimeScript RT reagent Kit (Takara Bio Inc., Shiga, Japan). Real-time PCR was performed with SYBR (Applied Biosystems, Foster City, CA, USA) using the ABI Prism 7700 sequence detection system (Applied Biosystems, Foster City, CA, USA). Data were collected and analyzed using the ABI Prism software (Applied Biosystems, Foster City, CA, USA). After detection of the threshold cycle for each mRNA in each sample, relative concentrations were calculated and normalized to β-actin. PCR conditions included an initial holding period at 95°C for 30 s, followed by a 2-step PCR program consisting of 95°C for 5 s and 60°C for 34 s for 40 cycles. All reactions were performed in duplicate. The following mouse primers were used (f, forward; r, reverse: Takara Bio Inc. Shiga, Japan): β-actin, IL-1β, IL-10, CCL2, TGF-β, Col1α1, MMP-9, MMP-13, and Gpnmb (Table 1).

**Table 1:** Primer lists.

gene	forward	reverse
IL-1β	TCCAGGATGAGGACATGAGCAC	GAACGTCACACACCAGCAGGTTA
IL-10	GACCAGCTGGACAACATACTGCTAA	GATAAGGCTTGGCAACCCAAGTAA
CCL2	GCATCCACGTGTTGGCTCA	CTCCAGCCTACTCATTGGGATCA
TGF-β	GTGTGGAGCAACATGTGGAACTCTA	TTGGTTCAGCCACTGCCGTA
Col1α1	GACATGTTCAGCTTTGTGGACCTC	GGGACCCTTAGGCCATTGTGTA
MMP-9	GCCCTGGAACTCACACGACA	TTGGAAACTCACACGCCAGAAG
MMP-13	TCCCTGGAATTGGCAACAAAG	GCATGACTCTCACAATGCGATTAC
Gpnmb	TCTGAACCGAGCCCTGACATC	AGCAGTAGCGGCCATGTGAAG
β-actin	CATCCGTAAAGACCTCTATGCCAAC	ATGGAGCCACCGATCCACA

### Western blot analysis

Liver tissues from mice were lysed with T-PER tissue protein extraction reagent (Pierce, Rockford, IL, USA) supplemented with protease inhibitor (Roche Diagnostics, Basel, Switzerland). Equal amounts of cell lysates (10 μg) were separated on 10% SDS polyacrylamide gels (SDS-PAGE) and electroblotted onto polyvinylidene fluoride membranes. The blots were probed with antibodies specific for anti-Gpnmb antibody (R&D Systems), β-actin (SantaCruz Biotechnology, Inc., Dallas, Texas, USA). After incubating the membrane with horseradish peroxidase−conjugated secondary antibodies, reactivity was visualized using electrogenerated chemiluminescence Western blotting detection reagents (GE Healthcare, Tokyo, Japan) and a Chemi Doc XRS-J digital densitometer (Bio-Rad Laboratories, Hercules, CA, USA).

### Isolation of Liver mononuclear cells (LMNCs) and Hepatic macrophages

After C57BL/6, DBA or DBA-g+ mice were treated with IP injections of CCl_4_, LMNCs were isolated as previously described [27, 28]. Under anesthesia, livers were perfused with Hanks balanced salt solution (HBSS; Life Technologies, Carlsbad, CA, USA) via the portal vein. After perfusion, livers were removed and minced with scissors. After the addition of 10 ml HBSS containing 0.05% collagenase (Wako, Osaka, Japan) and 0.01% DNase I (Roche Diagnostics), the specimens were shaken for 40 min at 37°C. Liver specimens were filtered through 100 μm cell strainers, suspended in 33% Percoll solution containing 10 U/ml heparin, and centrifuged for 15 min at 500g at room temperature. Non-parenchymal cell–enriched supernatant was collected and the cells were washed twice. Thus, LMNCs were purified. Additionally, isolated LMNCs were plated on non-collagen-coated plates for 30 min and washed with PBS three times as previously described [28]. More than 90% of attached cells were positive for both CD68 and F4/80. Therefore, these cells were considered to be hepatic macrophages. The viability of the isolated macrophages was more than 95% as determined by trypan blue exclusion.

### Flow Cytometry

LMNCs were prepared as previously described. After Fc receptor blockade, the cells were stained with APC-conjugated anti-F4/80 (Clone: BM8, eBioscience, San Diego, CA, USA), FITC-conjugated anti-CD11b (Clone: M1/70, eBioscience), and PE-conjugated anti-CD68 (Clone: FA-11, AbD Serotec, Raleigh, NC, USA). Alternatively, the cells were stained with FITC-conjugated anti-CD11b (Clone: M1/70, eBioscience), PE-conjugated anti-CD68 (Clone: FA-11, AbD Serotec), and anti-Gpnmb (Clone: #297310, R&D systems) followed by the addition of APC-conjugated secondary antibodies. Samples were evaluated using a CyAn ADP flow cytometer (Beckman Coulter, USA) and analyzed with Kaluza software (Beckman Coulter).

### Phagocytosis assay with microspheres

LMNCs (5X10^5^ cells/200 μl) were incubated with 3 μl FITC-microspheres (1X10^8^) for 20 min [27]. Thereafter, the cells were stained with PE-conjugated anti-CD11b (Clone: M1/70, eBioscience) or PE-conjugated anti-CD68 (Clone: FA-11, AbD Serotec), and anti-Gpnmb (Clone: #297310, R&D Systems) followed by the addition of APC-conjugated secondary antibodies. Samples were evaluated using a CyAn ADP flow cytometer (Beckman Coulter) and analyzed with Kaluza software (Beckman Coulter).

### Cell culture and transfection with siGpnmb

Hepatic macrophages were isolated as previously described. Attached hepatic macrophages were detached by incubation with TrypLE^™^ Select (Life Technologies, Carlsbad, USA) for 5 min. Cells were seeded in 12-well plates (2X10^5^ cells per well) followed by the addition of 5X10^5^ dead hepatocytes or control medium. The methods for the preparation of dead hepatocytes were as follows. After primary murine hepatocytes were isolated by liver perfusion, they were cultured overnight on collagen-coated dishes in Williams E medium (Life Technologies) supplemented with 10% FCS and 1% Pen/Strep solution. Adherent viable hepatocytes were detached using trypsin, washed, and cultured in suspension in serum-free Williams E medium containing anti-mouse CD95 (BD Pharmingen, Franklin Lakes, NJ, USA) at 37°C for 8 h [29]. To inhibit Gpnmb mRNA, hepatic macrophages were seeded in 12-well plates (2X10^5^ cells per well) and cultured in RPMI 1640 (Life Technologies) supplemented with 10% FBS (Medical & Biological Laboratories, Nagoya, Japan) for 12 h. These cells were transfected with siRNA specific for Gpnmb (UUAGCAUCUUCCUUCUGGCAUCUGG) (Invitrogen) using RNAiMAX (Invitrogen) according to the manufacturer’s instructions. Stealth RNAi Negative Control Medium GC Duplexes (Invitrogen) were used as negative control siRNA.

### ELISA for TGF-Β and MMP-13

Supernatants of hepatic macrophages added to dead hepatocytes and transfected with siGpnmb were concentrated using Amicon Ultra (Merck Millipore). Thereafter, the protein concentrations of concentrated supernatants were measured with Nano Drop 2000 (Thermo Fisher Scientific, Inc.), and TGF-β or MMP-13 concentrations were measured with an ELISA kit (R&D Systems)

### Statistical analysis

All data were expressed as means and standard errors of the mean. Data between groups were analyzed by Mann-Whitney U test. Differences between multiple groups were compared with one-way analysis of variance (SPSS version 15.0). P values less than 0.05 were considered statistically significant.

## Results

### Macrophages infiltrate into injured livers during the repair process after a single injection of CCl_4_

**Fig 1:**
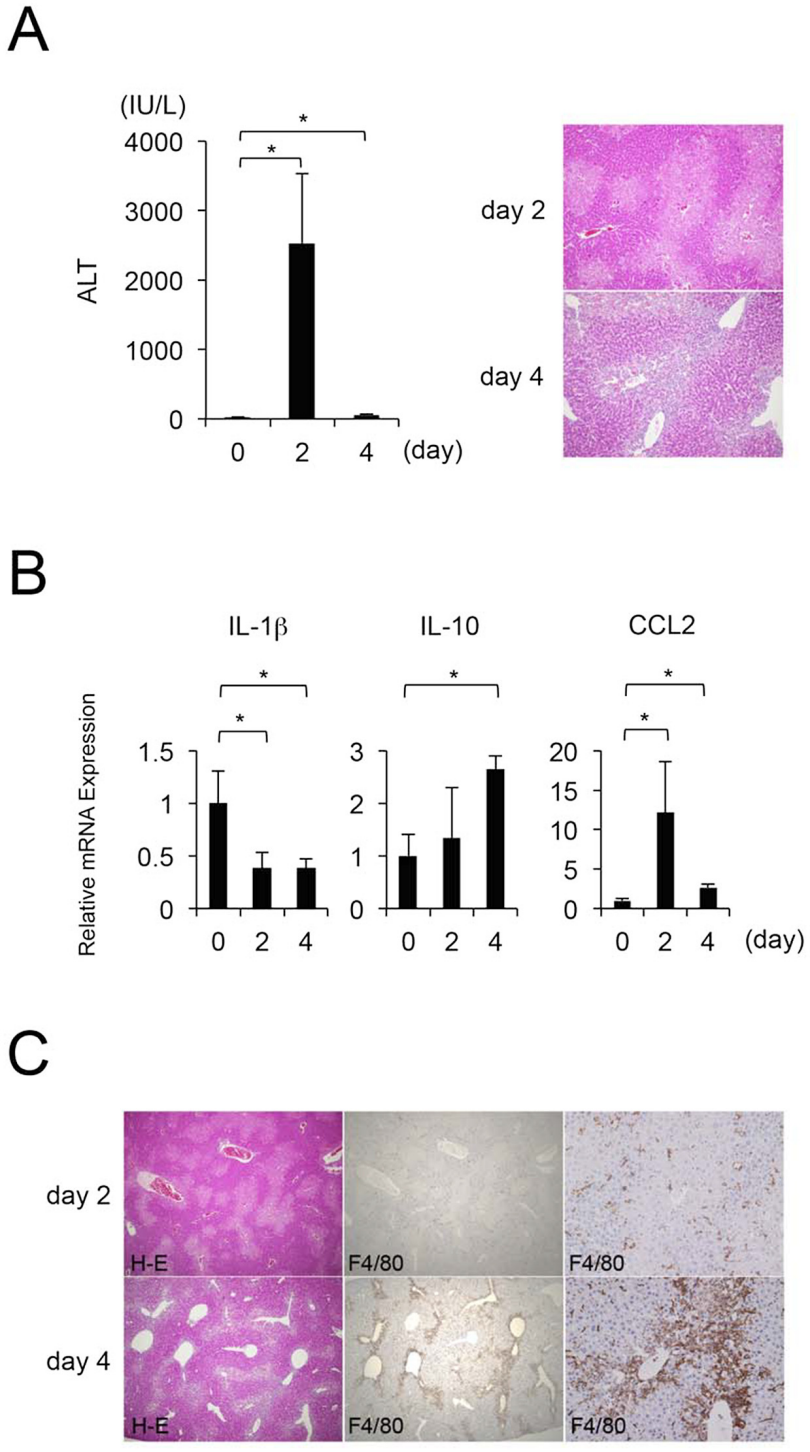
Sequential changes of ALT and histological examination in ccl_4_-induced acute liver injury. At four days after CCl_4_ injection, (A) Serum ALT decreases and centrilobular necrosis are reduced (original magnification, x100). Values are mean ± SEM (n = 4). * *P* < 0.05 vs. the 0^th^ day (Tukey’s HSD test). (B) Hepatic IL-1β, IL-10 and CCL2 expression reveals that at the 4^th^ day after CCl_4_ injection the repair process is in the recovery phase. Values are mean ± SEM (*n* = 4). * *P* < 0.05 vs. the 0^th^ day (Tukey’s HSD test). (C) Hepatic macrophages infiltrating into the liver injury (original magnification, x40 and x200).

First, we examined sequential changes in parameters of hepatic injury induced by a single injection of CCl_4_. Serum ALT levels significantly increased, and centrilobular necrosis developed two days after CCl_4_ injection, followed by a decrease in both serum ALT and the area of centrilobular necrosis (Fig 1A). Although the expression of IL-1β in liver tissues was decreased at two and four days after CCl_4_ injection, IL-10 expression gradually increased after CCl_4_ injection (Fig 1B). Immunohistochemically, marked infiltration of macrophages positive for F4/80 around the necrotic areas was observed at four days, but not two days, after CCl_4_ injection (Fig 1C).

### CCl_4_-induced liver injury persists when infiltrating macrophages are depleted

**Fig 2:**
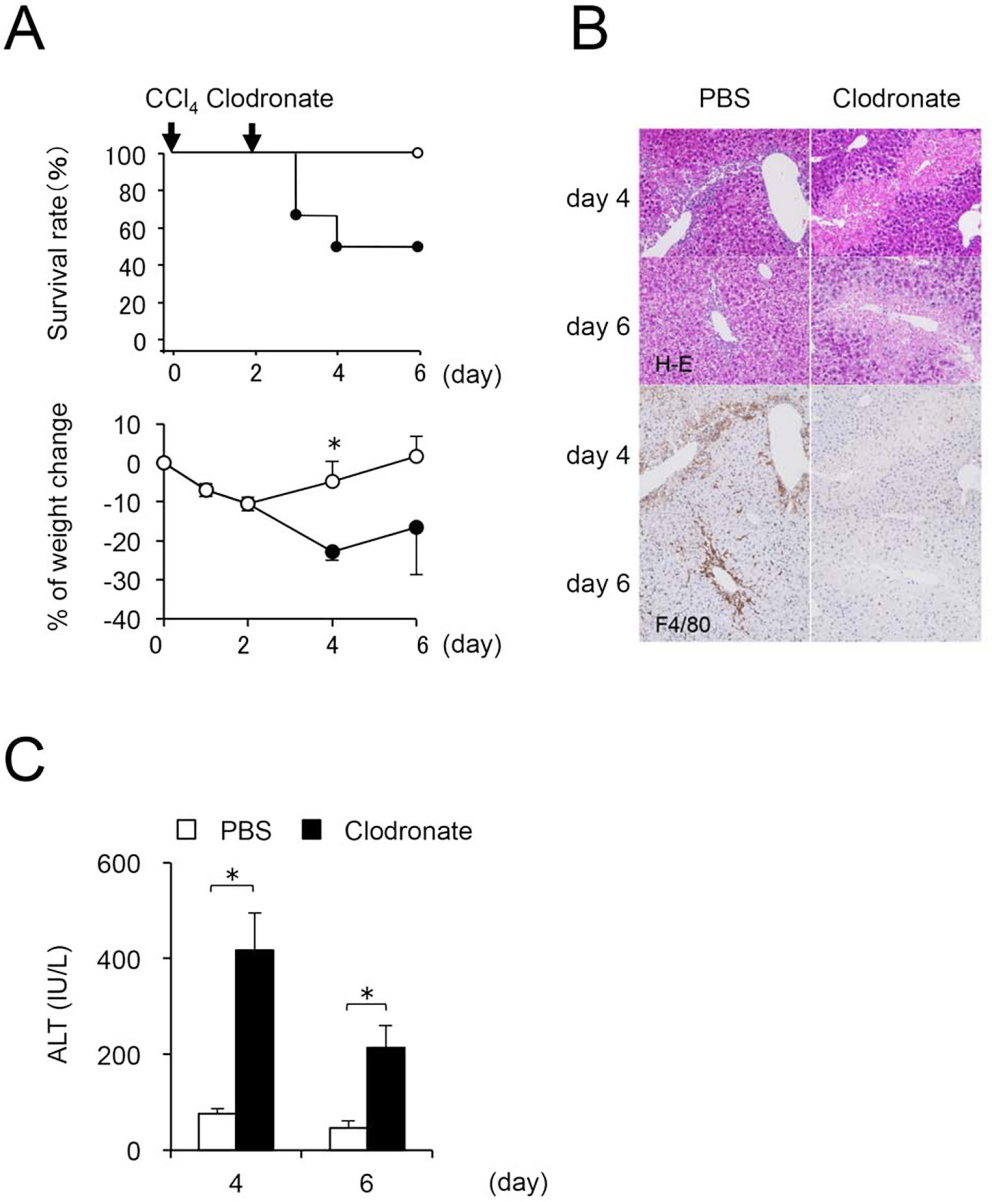
Impact on liver injury by depletion of infiltrating hepatic macrophages during the recovery phase. After clodronate liposome injection at the 2^nd^ day after CCl_4_ injection (*n* = 6 vs. PBS injection group (*n* = 4)), (A) the survival rate is decreased and weight loss is remarkable. (B) Centrilobular necrosis persists (original magnification, x200) and (C) serum ALT levels increase significantly (clodronate liposome injection group (*n* = 3 (only survival)) vs. PBS injection group (*n* = 4)). Values are mean ± SEM. * *P* < 0.05 (Mann-Whitney U test)).

To elucidate the role of macrophages during the repair process in injured livers, infiltrating macrophages were depleted by treatment with clodronate liposomes at two days after a CCl_4_ injection. Although all animals treated with CCl_4_ alone survived for the duration of the experimental period (six days), animals injected with clodronate liposomes exhibited a significant decrease in body weight, and 50% of animals died at four days after CCl_4_ injection (Fig 2A). Treatment with clodronate liposomes abolished macrophage infiltration at four and six days after CCl_4_ injection (Fig 2B), and a sustained increase in serum ALT and persistent centrilobular necrosis were observed (Fig 2B and 2C).

**Fig 3:**
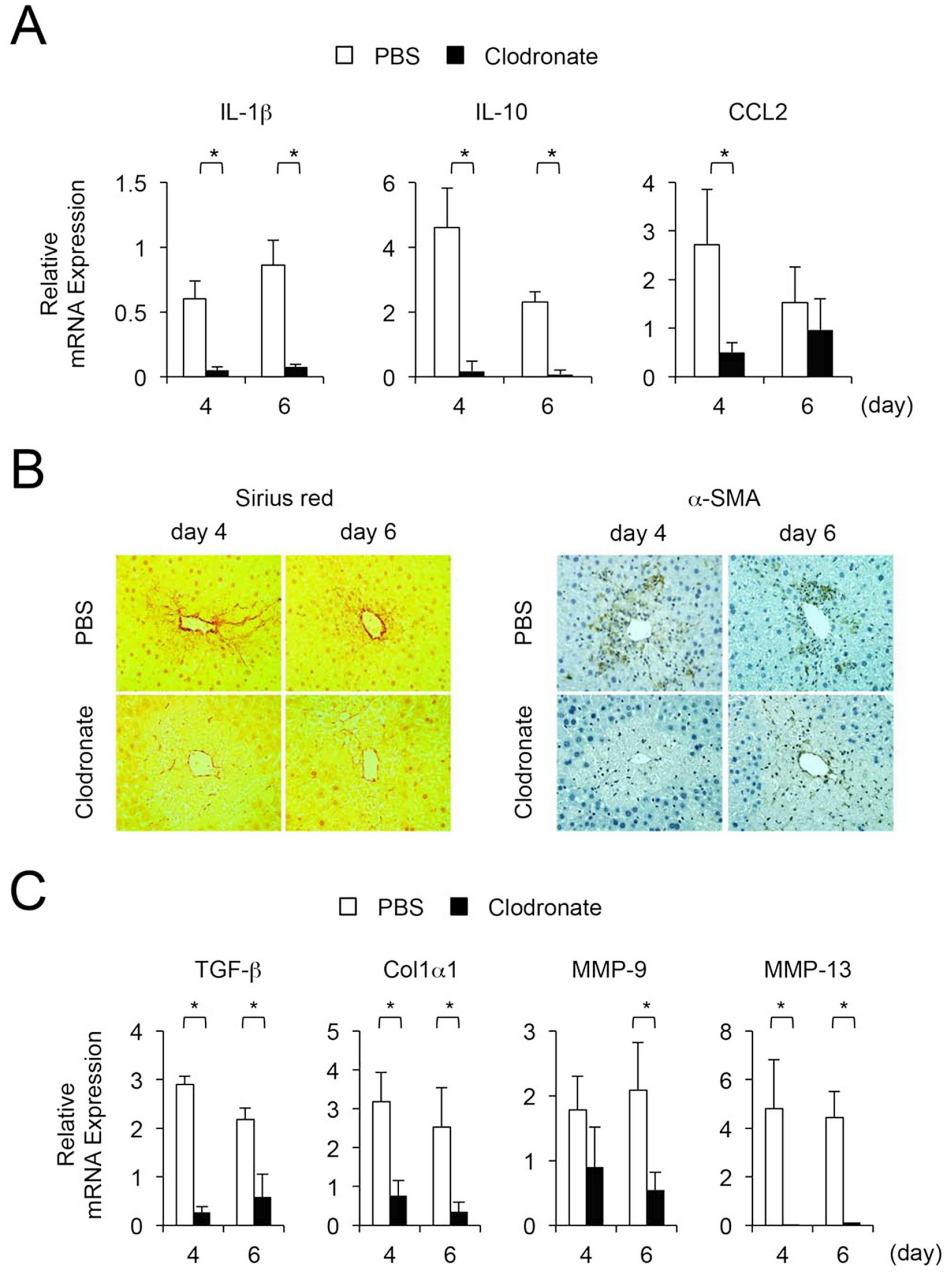
Impact on liver injury repair process by depletion of infiltrating hepatic macrophages during the recovery phase. (A) Hepatic expression of IL-1β, IL-10, and CCL2 are significantly decreased (clodronate liposome injection group [*n* = 3, only survival] vs. PBS injection group [*n* = 4)]). Values are mean ± SEM. * *P* < 0.05 (Mann-Whitney U test)). (B) Collagen deposition and α-SMA expression are decreased (original magnification, x400). (C) Hepatic expression of TGF-β, Col1α1, and MMP-13 are significantly decreased (clodronate liposome injection group [*n* = 3, only survival] vs. PBS injection group [*n* = 4]). Values are mean ± SEM. * *P* < 0.05 (Mann-Whitney U test)).

To evaluate the repair process following liver injury, we examined the expression of cytokines and collagen deposition (clodronate liposome injection group, n = 3 [only survival]; PBS injection group, n = 4). Expression of IL-1β, IL-10 and TGF-β in liver tissues was significantly decreased by an injection of clodronate liposomes (Fig 3A). Additionally, collagen deposition and α-SMA staining were diminished (Fig 3B), and expression of Col1α1 and MMP-13 was significantly reduced by this treatment (Fig 3C). These results suggest that depletion of infiltrating macrophages inhibits the repair of liver injury through lack of pro- and anti-inflammatory cytokine production and collagen deposition.

### Gpnmb is expressed in CD68-positive macrophages that infiltrate injured livers, and Gpnmb-positive macrophages show enhanced phagocytic activity

**Fig 4:**
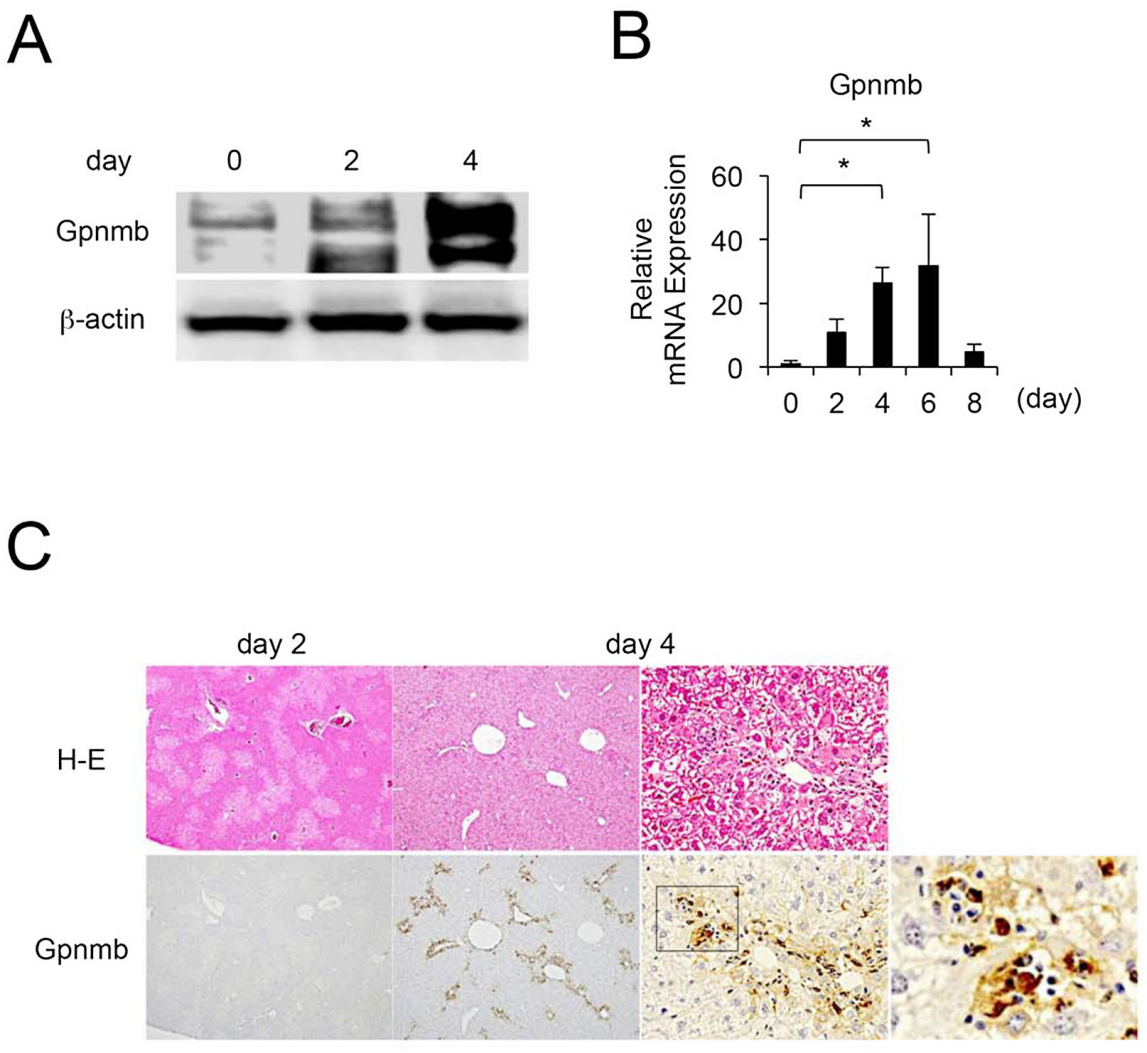
Sequential changes in and localization of Gpnmb expression. Gpnmb expression is enhanced in the recovery phase (A) in whole liver as determined by Western blotting and (B) in isolated hepatic macrophages as determined by quantitative real-time polymerase chain reaction (*n* = 5 at each day). (C) Moreover, Gpnmb expression is observed immunohistochemically around injured lesions in a pattern similar to that in F4/80-positive macrophages, and Gpnmb-positive cells exhibit partial phagocytosis.

Since a previous report showed that Gpnmb was expressed in hepatic macrophages in rats with acute liver injury induced by single CCl_4_ injections [18], we further examined sequential changes in Gpnmb expression in liver tissues and infiltrating macrophages (*N* = 5 at each day). Expression of Gpnmb in liver tissues was stimulated at two and four days after a single injection of CCl_4_ (Fig 4A). Also, Gpnmb expression in hepatic macrophages isolated from injured liver tissues gradually increased, peaking at four and six days after a CCl_4_ injection (Fig 4B). Immunohistochemically, Gpnmb-positive cells were observed around the necrotic areas, similar to F4/80-positive macrophages (Fig 1C), and also exhibited phagocytosis (Fig 4C).

**Fig 5:**
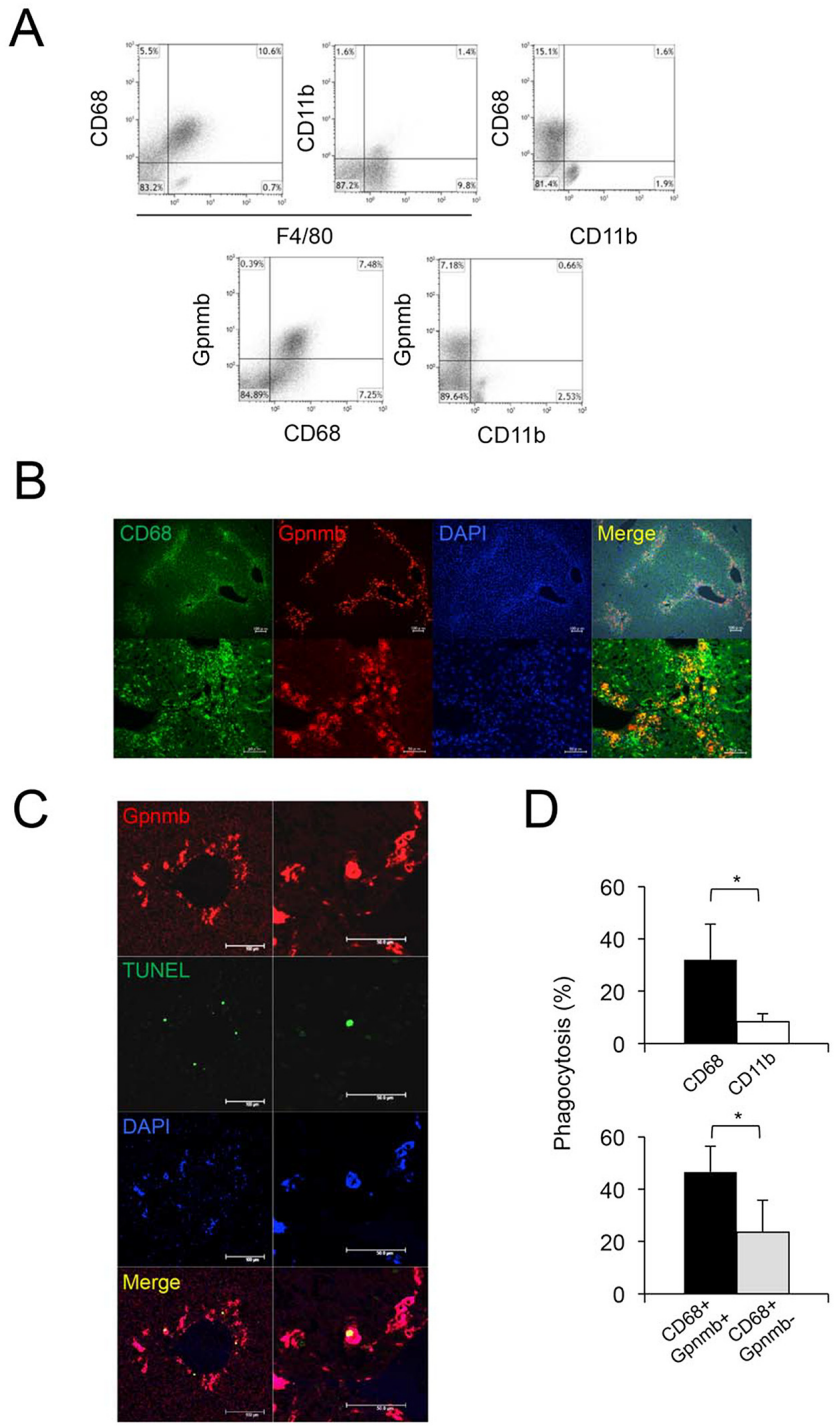
Characteristics of gpnmb-positive cells infiltrating liver tissue. In LMNCs isolated from injured liver, (A) more than 90% of hepatic macrophages positive for F4/80 are also positive for CD68, whereas CD11b is expressed in approximately 10% of F4/80-positive cells. Gpnmb expression is detected in approximately 50% of CD68-positive cells. (B) Immunofluorescent double staining shows that some CD68-positive cells are also positive for Gpnmb (scale bar: upper, 100 μm; lower, 50 μ m). (C) Some Gpnmb-positive cells show phagocytosis of apoptotic cells (scale bar: left side, 100 μm; right side, 50 μm). (D) Phagocytic activity of CD68-positive cells is significantly higher than that of CD11b-positive cells. Additionally, in CD68-positive cells, phagocytic activity of Gpnmb-positive cells is significantly increased compared with that in Gpnmb-negative cells. Values are mean ± SEM (*n* = 3). * *P* < 0.05 (Mann-Whitney U test).

To investigate the phenotype of Gpnmb-positive cells, we performed flow cytometry on LMNCs isolated at four days after CCl_4_ injection. More than 90% of hepatic macrophages positive for F4/80 were also positive for CD68, whereas CD11b was expressed in approximately 10% of F4/80-positive cells (Fig 5A). Gpnmb expression was detected in approximately 50% of CD68-positive cells, whereas only a few CD11b-positive cells expressed Gpnmb. Immunofluorescent double staining showed that some CD68-positive cells were also positive for Gpnmb (Fig 5B).

Recently, Kinoshita et al. reported that CD68-positive and CD11b-positive hepatic macrophages infiltrating injured liver tissues exhibited predominantly phagocytosis and cytokine production, respectively [27]. Since some Gpnmb-positive cells showed phagocytosis of apoptotic cells (Fig 5C), we examined the phagocytic activity of isolated LMNCs. The phagocytic activity of CD68-positive cells was significantly greater than that of CD11b-positive cells (Fig 5D). Additionally, among CD68-positive cells, the phagocytic activity of Gpnmb-positive cells was significantly greater than that of Gpnmb-negative cells.

### Lack of macrophages positive for Gpnmb decreases collagen deposition following liver injury via decreased expression of MMP-9, MMP-13 and TIMP-1

**Fig 6:**
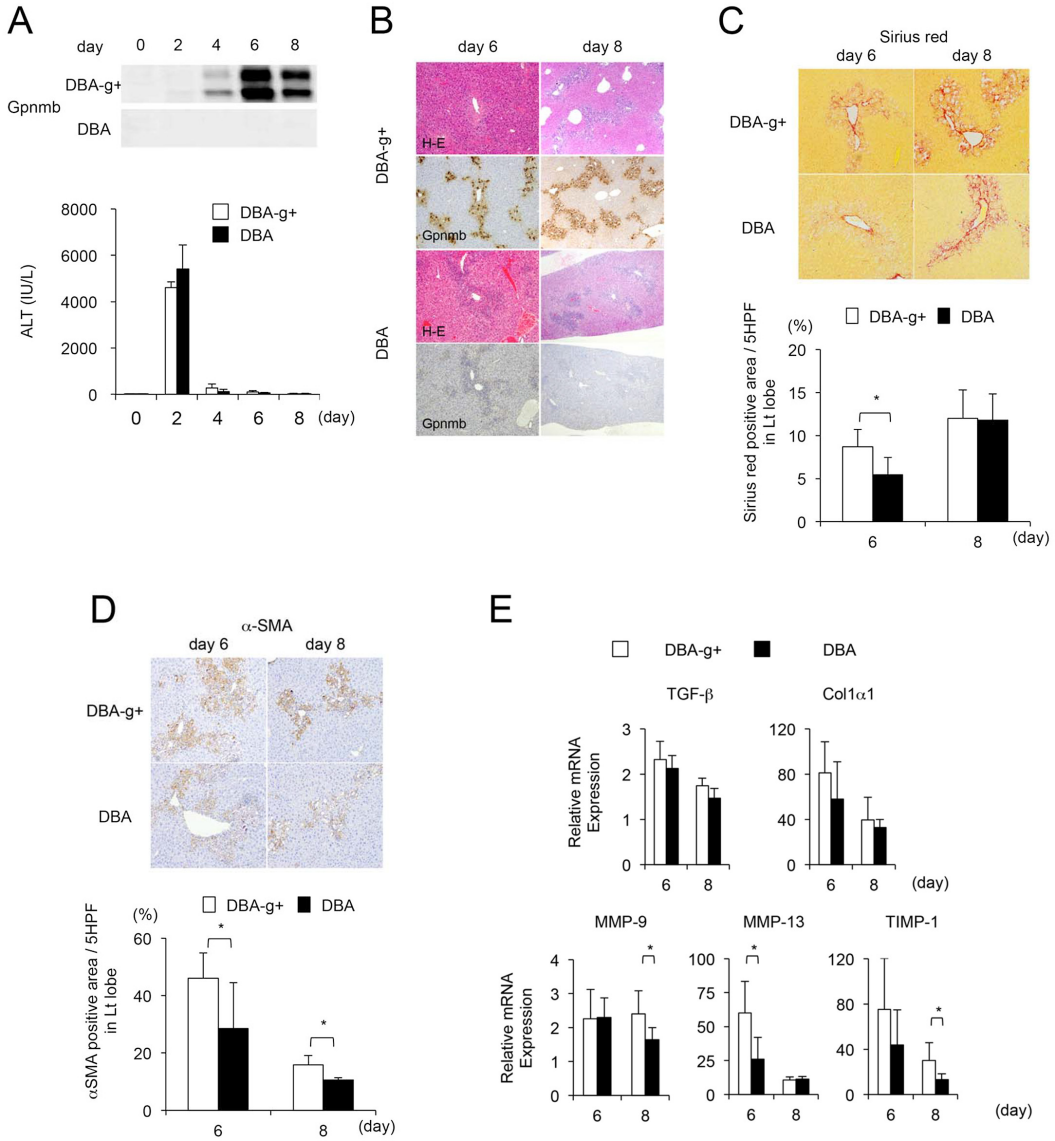
Features of DBA-g+ and DBA mice. (A) Expression of Gpnmb is observed in DBA-g+ but not DBA mice following a single injection of CCl_4_. However, (B) sequential changes in serum ALT levels and the degree of liver injury are not affected by the lack of Gpnmb-positive macrophages (original magnification, x100). Conversely, (C) the areas of fibrosis and (D) the number of α-SMA-positive cells are significantly decreased in the liver tissues of DBA mice compared to DBA-g+ mice (original magnification, x200). Additionally, (E) although lack of Gpnmb-positive macrophages does not affect expression of TGF-β or Col1α1, expression of MMP-9, MMP-13, and TIMP-1 are significantly decreased in mice lacking Gpnmb expression at six or eight days after single injection of CCl_4_. Values are mean ± SEM (*n* = 4). * *P* < 0.05 (Mann-Whitney U test).

To further investigate the role of Gpnmb-positive macrophages that have infiltrated into injured livers, we induced CCl_4_-induced liver injuries in DBA and DBA-g+ mice (Fig 6). Following a single injection of CCl_4_, Gpnmb expression was observed in DBA-g+ but not DBA mice (Fig 6A). However, sequential changes in serum ALT levels and the degree of liver injury were not affected by lack of Gpnmb-positive macrophages (Fig 6A and 6B). Conversely, the areas of fibrosis and α -SMA-positive cells were significantly decreased in the liver tissues of DBA mice in comparison with DBA-g+ mice (Fig 6C and 6D). Additionally, although lack of Gpnmb-positive macrophages did not affect the expression of TGF-β or Col1α1, the expression of MMP-9, MMP-13 and TIMP-1 was significantly decreased in mice lacking Gpnmb expression at six or eight days after a single injection of CCl_4_ (Fig 6E).

### Phagocytosis stimulates secretion of MMP-13, whereas Gpnmb is involved in MMP-13 but not TGF- β secretion

**Fig 7:**
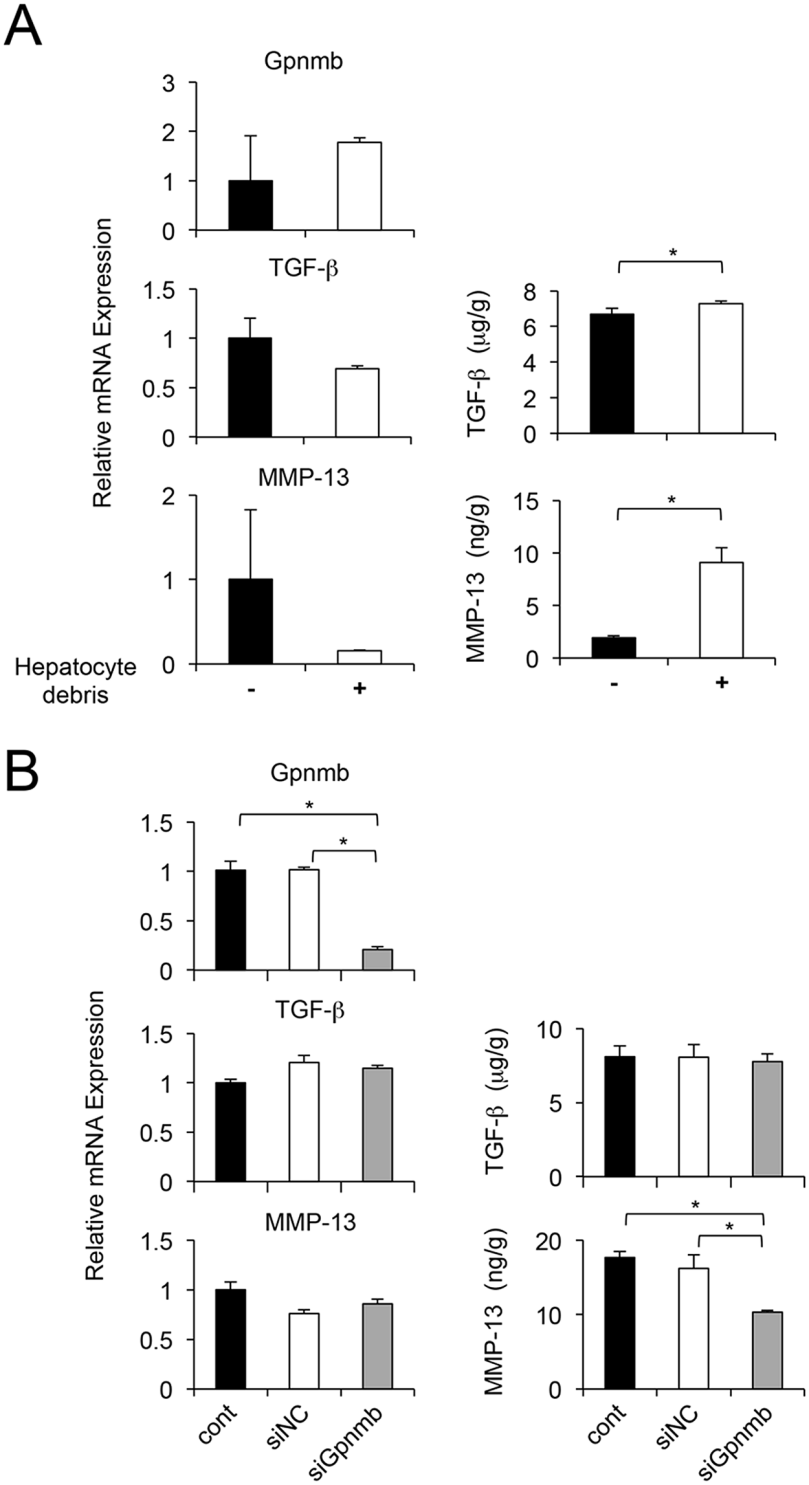
Effects of Gpnmb expression on hepatic macrophages. In infiltrating hepatic macrophages isolated from injured livers, (A) co-culture with apoptotic hepatocytes does not affect mRNA expression of Gpnmb, TGF-β, or MMP-13, but does increase TGF-β and MMP-13 secretion. Values are mean ± SEM (*n* = 3). * *P* < 0.05 (Mann-Whitney U test). Moreover, (B) mRNA expression of TGF-β and MMP-13 is not affected by inhibited Gpnmb expression, however secretion of MMP-13, but not TGF-β is significantly decreased by inhibition of Gpnmb expression. Values are mean ± SEM (*n* = 3). * *P* < 0.05 vs. control (Tukey’s HSD test).

We isolated LMNCs from C57BL6 mice at four days after a single injection of CCl_4_, and investigated the effect of phagocytosis and Gpnmb expression on MMP-13 production. Although co-culture with apoptotic hepatocytes did not affect the mRNA expression of Gpnmb, TGF-βor MMP-13, engulfing hepatocyte debris significantly increased the secretion of TGF-β and MMP-13 (Fig 7A). Conversely, mRNA expression of TGF-β and MMP-13 was not affected by inhibited Gpnmb expression. However, the secretion of MMP-13, but not TGF-β was significantly decreased by inhibition of Gpnmb expression (Fig 7B).

## Discussion

The liver has an enormous capacity to regenerate, sufficient to recover its original mass and function even after 70% partial hepatectomy. The recovery process requires a dynamic interplay between parenchymal and non-parenchymal cells. In the early phase of liver regeneration, corresponding with hepatocyte proliferation, activated macrophages produce TNF-α and IL-6, which are essential for the proliferation of mature hepatocytes [30]. However, the role of infiltrating hepatic macrophages in the later phase of liver regeneration has thus far remained unclear. In this study, we first examined the involvement of hepatic macrophages in mice with CCl_4_-induced acute liver injury. In the injured liver tissues, hepatic CCL2 expression increased two days after CCl_4_ injection, and marked infiltration of F4/80-positive macrophages around necrotic areas was observed four days after CCl_4_ injection. In comparison to liver tissues at two days after CCl_4_ injection, by four days the necrotic areas were reduced in size and the expression of inflammatory cytokines had decreased, while the expression of anti-inflammatory cytokines had increased. Moreover, when infiltrating macrophages were depleted by clodronate liposomes at two days after CCl_4_ injection, liver injury was sustained at four days after CCl_4_ injection. These results indicate that macrophages infiltrating injured liver tissues at four days after CCl_4_ injection possibly diminish the inflammatory response and play a pivotal role in the repair of injured livers.

Recently, Kinoshita et al. reported that both CD11b-positive macrophages, which produce primarily inflammatory cytokines, and CD68-positive macrophages, which exhibit phagocytic activity, infiltrated injured livers in an animal model of lipopolysaccharide-induced liver injury [27]. In the current study, approximately 95% of infiltrating macrophages isolated from injured liver tissues four days after CCl_4_ injection were positive for CD68, and CD68-positive macrophages exhibited high phagocytic activity compared with CD11b-positive macrophages. Tissue repair processes are divided into three phases: the inflammatory phase, proliferation phase, and remodeling phase. The inflammatory phase consists of inflammatory cell accumulation, proinflammatory cytokine production and phagocytosis of necrotic debris. The proliferative phase is characterized by cell proliferation, angiogenesis and collagen production. The remodeling phase involves maturation of the regenerated or repaired tissue and resolution of fibrosis [31–33]. These phases overlap during the repair process. Phagocytosis, an important role of macrophages, is observed from the inflammatory phase to the proliferative phase; macrophage phagocytosis induces either repression or promotion of inflammation, and also either fibrosis development or resolution [34]. Thus, both development and resolution of fibrosis are important components of the repair process in injured livers, and macrophages play critical roles in these processes. In this study, depletion of infiltrating macrophages during the recovery phase of CCl_4_-induced liver injury suppressed collagen production and activation of hepatic stellate cells. These results are supported by previous investigations showing that suppression of macrophage infiltration inhibited activation of hepatic stellate cells and liver fibrogenesis in rats with dimethylnitrosamine-induced hepatic fibrosis [35]. Conversely, in an animal model of liver cirrhosis, macrophages were shown to play both profibrogenic and antifibrotic roles, in the inflammatory and recovery phases, respectively [11]. In this study, we investigated a mouse model of acute liver injury induced by a single injection of CCl_4_, and showed that depletion of macrophages decreased not only TGF-β and Col1α1 expression but also expression of MMP-13, despite a decrease in collagen production. Recently, Dechêne et al. demonstrated that patients with acute liver failure exhibited increased liver stiffness and serum TIMP-1 levels, and also showed a simultaneous increase in serum levels of MMP-2 [36]. We speculated that infiltrating hepatic macrophages play an important role in both fibrosis development and resolution, and that these processes are required for the repair of injured livers.

Gpnmb is expressed in infiltrating hepatic macrophages in mice with CCl_4_-induced acute liver injury, while the role of macrophages positive for Gpnmb is unknown. In this study, Gpnmb was expressed in approximately half of CD68-positive macrophages infiltrating injured liver tissues. Moreover, CD68-positive and Gpnmb-positive macrophages exhibited enhanced phagocytic activity compared to CD68-positive and Gpnmb-negative macrophages. Recent investigations have reported that Gpnmb is required for apoptotic cell clearance in kidneys subjected to ischemia reperfusion injury [37], and that Gpnmb acts as a negative regulator of macrophage inflammatory responses [20]. Therefore, we investigated the effect of Gpnmb on CCl_4_-induced liver injury using DBA mice, which do not express Gpnmb. However, when compared to DBA-g+ mice, sequential changes in serum ALT levels and the degree of liver injury were not affected by the lack of Gpnmb-positive macrophages. Nonetheless, the areas of fibrosis and α-SMA-positive cells were significantly decreased in liver tissues of DBA mice in comparison with DBA-g+ mice. Additionally, the lack of Gpnmb-positive macrophages resulted in significantly decreased expression of MMP-9, MMP-13, and TIMP-1 at six or eight days after a single injection of CCl_4_. Once liver injury occurs, macrophages and hepatic stellate cells phagocytose necrotic and apoptotic cells, and activated macrophages and hepatic stellate cells secrete proinflammatory and profibrotic mediators. The secretion of TGF-β then leads to transdifferentiation of hepatic stellate cells into myofibroblasts. Hepatic stellate cell-derived myofibroblasts express α-SMA and produce collagen [38], and the collagen tissues protect hepatocytes against various toxic stimuli [39]. Finally, the balance of MMP and TIMP secretion by myofibroblasts and macrophages ensure a constant turnover of the matrix, and injured liver tissues are repaired. In this study, the phagocytic stimulus for isolated infiltrating hepatic macrophages at four days after CCl_4_ injection did not affect Gpnmb expression. However, the phagocytic stimulus and Gpnmb expression each individually increased MMP-13 secretion in isolated infiltrating hepatic macrophages. Recently, several investigations have shown that Gpnmb accelerates the motility of hepatoma cells [24] and promotes breast cancer metastasis to bone by inducing MMP-3 expression [40]. Additionally, Gpnmb upregulates expression of MMP-3 and MMP-9 in fibroblasts [41–42]. Conversely, MMP-13 has antifibrotic effects in some contexts but also functions as a profibrotic molecule in cholestatic livers [43], and plays an important role in the maturation of myofibroblasts [44]. Therefore, Gpnmb-positive macrophages showing enhanced phagocytic activity are closely associated with the activation of hepatic stellate cells via MMP-13 secretion, and consequently play an important role in repair process following liver injury.

A main limitation of our study was that it used only one model of acute liver injury, namely that induced by CCl_4_. Our experimental model may reflect one role of Gpnmb in acute liver injury. However, we need to confirm the role of Gpnmb in other liver injury models such as those involving chronic liver injury. Moreover, we are interested in subpopulations of Gpnmb-expressing macrophages such as M1 or M2, and whether Gpnmb might influence the differentiation from M1 to M2 macrophages. We, therefore, examined expression of M1 and M2 markers in thioglycolate-elicited peritoneal macrophages isolated from DBA and DBA-g+ mice (S1 Fig); expression of Gpnmb was detected in approximately 20% of peritoneal macrophages of DBA-g+ mice (data not shown), whereas macrophages of DBA mice did not express Gpnmb. Expression of M2 markers, Mrc-1 and FIZZ1, significantly increased in macrophages isolated from DBA-g+ mice, when compared with those from DBA mice. However, not only a M1 marker, iNOS, but also other M2 markers, Arg-1 and YM-1, were equally expressed in macrophages from both mice. These results suggest that a subpopulation of Gpnmb-positive macrophages consist of diverse phenotypes of macrophages, and further experiments are required to clarify detailed functions of Gpnmb expressed in infiltrating hepatic macrophages during the healing process of injured livers.

In conclusion, Gpnmb was expressed in infiltrating hepatic macrophages positive for CD68, and Gpnmb-positive macrophages exhibited enhanced phagocytic activity and contributed to the repair of acute liver injury through MMP-13 secretion. Although further investigations are required to clarify the exact role of Gpnmb in macrophages, the findings presented here shed light on the complex mechanisms involved in the repair of liver injury.

## Supporting Information

S1 Fig M1/M2 markers in thioglycolate-elicited peritoneal macrophages isolated from DBA-g+ and DBA mice. Values are mean ± SEM (*n* = 3). ** *P* < 0.001 (Mann-Whitney U test).
(EPS)

## Abbreviations

Gpnmb: Glycoprotein nonmetastatic melanoma protein B
OA: osteoactivin
MMP: matrix metalloproteinase
IL: interleukin
LMNCs: Liver mononuclear cells
α-SMA: alpha-smooth muscle actin
CCl_4_: carbon tetrachloride
CCL2: chemokine (C-C motif) ligand 2
TGF-β: transforming growth factor-β
Col1α1: collagen type 1 alpha 1
TIMP: tissue inhibitor of metalloproteinases
PCR: polymerase chain reaction
